# A randomised pilot Phase II study of doxorubicin and cyclophosphamide (AC) or epirubicin and cyclophosphamide (EC) given 2 weekly with pegfilgrastim (accelerated) *vs* 3 weekly (standard) for women with early breast cancer

**DOI:** 10.1038/sj.bjc.6604862

**Published:** 2009-01-22

**Authors:** R L Jones, G Walsh, S Ashley, S Chua, R Agarwal, M O'Brien, S Johnston, I E Smith

**Affiliations:** 1 Breast Unit, Royal Marsden NHS Trust, Fulham Road, London SW3 6JJ, UK; 2 Institute of Cancer Research, Fulham Road, London, UK

**Keywords:** early breast cancer, doxorubicin, epirubicin, accelerated/dose-dense chemotherapy

## Abstract

Accelerated (dose-dense) chemotherapy, in which the frequency of administration is increased without changing total dose or duration, may increase the efficacy of cancer chemotherapy. We performed a randomised Phase II study to assess the safety and relative toxicity of AC (doxorubicin; cyclophosphamide) *vs* E(epirubicin)C given by conventional or accelerated schedules as neoadjuvant or adjuvant chemotherapy for early breast cancer. Furthermore, the relative toxicity of doxorubicin and epirubicin remains uncertain. Patients were randomised to one of four arms; four courses of standard 3 weekly cyclophosphamide 600 mg m^−2^ in combination with doxorubicin 60 mg m^−2^ (AC) *vs* epirubicin 90 mg m^−2^ (EC) 3 weekly *vs* the same regimens administered every 2 weeks with pegfilgrastim (G-CSF). A total of 126 patients were treated, 42 with standard AC, 42 with accelerated AC, 19 with standard EC and 23 with accelerated EC. Significantly more grade 3/4 day one neutropenia was seen with standard (6/61, 10%) compared to accelerated (0/65,) regimens (*P*=0.01). A trend towards more neutropenic sepsis was seen in the combined standard and accelerated AC arms (12/84, 14%) compared to the combined EC arms (1/42, 2%), *P*=0.06. Falls in left ventricular ejection fraction were not increased with accelerated treatment. Accelerated AC and EC with pegfilgrastim are safe and feasible regimens in the treatment of early breast cancer with less neutropenia than conventional 3 weekly schedules.

Adjuvant anthracycline chemotherapy results in a significant survival benefit in women with moderate to high-risk early breast cancer (Early Breast Cancer Trialists' Collaborative Group, 2005). A large randomised trial has demonstrated four cycles of adjuvant doxorubicin and cyclophosphamide (AC) to be as effective as 6 cycles of CMF (cyclophosphamide, methotrexate and 5-FU) in women with node-positive disease (Fisher *et al*, 1990). Subsequently, AC has become widely used throughout the world. Adjuvant anthracycline-based schedules have frequently included epirubicin instead of doxorubicin, but no data exist directly comparing the efficacy and safety of these two anthracyclines in early breast cancer.

Taxanes given sequentially or concurrently with anthracyclines have resulted in a further small, but significant, improvement in disease-free and overall survival particularly in node-positive disease (Bria *et al*, 2006), but short duration anthracycline/cyclophosphamide schedules are still widely used for moderate risk patients.

A randomised trial of 2005 women has demonstrated a significant survival benefit for women, with lymph node-positive breast cancer, treated with dose-dense (2 weekly) chemotherapy with granulocyte colony stimulating factor (G-CSF) support compared to conventional 3 weekly schedules (Citron *et al*, 2003).

Our study aimed to assess the safety profile including cardiac safety of short duration (4 courses) standard AC *vs* EC given 3-weekly compared with accelerated 2-week schedules of these two regimens.

## Patients and methods

### Patient selection

Patients with early breast cancer, suitable for adjuvant chemotherapy, were recruited following Research Ethics Committee approval. Written informed consent was obtained before trial entry. Inclusion criteria were age less than 70 years; ECOG performance status 0–1; adequate haematological, renal and hepatic function; normal left ventricular ejection fraction (LVEF), assessed using multigated acquisition (MUGA) scan or echocardiography. Further inclusion criteria for the adjuvant arms were complete excision of histological confirmed adenocarcinoma; axillary resection or sentinel node biopsy; lymph node negative disease or 1–3 involved nodes if postmenopausal. Those entered into the neoadjuvant arms were required to have palpable tumours ⩾2 cm.

### Patient assessment

Initial assessment included history and physical examination, full blood count, routine biochemistry, hormone receptor status, chest X-ray, electrocardiogram and MUGA scan. For the neoadjuvant arms, bi-dimensional calliper tumour measurements were obtained before therapy and after each cycle as well as ultrasonography at baseline and following cycles 2 and 4.

### Treatment

Patients were randomly assigned to one of four arms:

Standard AC – four cycles of doxorubicin 60 mg m^−2^ and cyclophosphamide 600 mg m^−2^ on day 1, every 21 days;

Accelerated AC – four cycles of doxorubicin 60 mg m^−2^ and cyclophosphamide 600 mg m^−2^ on day 1, every 14 days with depot recombinant human G-CSF (pegfilgrastim) 6 mg subcutaneous injection on day 2 (Neulasta; Amgen, Cambridge, UK);

Standard EC – four cycles of epirubicin 90 mg m^−2^ and cyclophosphamide 600 mg m^−2^ on day 1, every 21 days;

Accelerated EC – four cycles of epirubicin 90 mg m^−2^ and cyclophosphamide 600 mg m^−2^ on day 1, every 14 days with pegfilgrastim on day 2.

Neoadjuvant patients were only randomised into the AC arms, because epirubicin was not recognised as a standard neoadjuvant therapy at the time of designing this trial.

Adjuvant endocrine therapy for a minimum of 5 years was given to those with hormone receptor-positive disease. Adjuvant radiotherapy was administered according to local policy at standard dose and fractionation.

### Response and toxicity assessment

Clinical response was assessed before each cycle of neoadjuvant chemotherapy using WHO criteria (Miller *et al*, 1981). A pathological complete response (pCR) was defined as complete disappearance of invasive cancer cells in breast and nodes on histological examination (Sataloff *et al*, 1995).

Toxicity for each cycle was recorded before the commencement of the following cycle and was evaluated using the National Cancer Institute Common Toxicity Criteria. Haematological toxicity was determined before each course (day 21 for standard and day 14 for accelerated regimens).

### End points

The primary end point was the incidence of grade 3/4 neutropenia on day 1 of each cycle. Secondary endpoints were to compare other toxicity including cardiotoxicity; compliance and feasibility; the percentage of intended dose given.

### Cardiotoxicity

Anthracycline cardiotoxicity was not anticipated with the cumulative doses used, but a protocol specification was included to identify any patient with cardiac failure and exclude them from further anthracycline-based therapy. Assessment of LVEF was performed at baseline, completion of chemotherapy, 1 and 2 years after chemotherapy.

### Dose modifications

Dose modifications were made for main toxicities as follows:

#### Myelosuppression

Chemotherapy was delayed by weekly intervals (maximum 3 weeks for 3 weekly and 2 weeks for 2 weekly regimens) until the neutrophil count ⩾1 × 10^9^ per litre and platelet count ⩾100 × 10^9^ per litre.

#### Febrile neutropenia

For standard arms, the dose of anthracycline and cyclophosphamide were reduced by 20% and G-CSF administered according to American Society of Clinical Oncology guidelines (Ozer *et al*, 2000). For accelerated arms, 20% dose reduction of anthracycline and cyclophosphamide was made in subsequent cycles.

### Statistical methods

To answer the trial aims, 82 evaluable patients were required to be randomised in the adjuvant arms. This component of the study was designed to have a 90% probability of detecting a 30% reduction in grade 3/4 neutropenia from 40% (AC/EC) to 10% (accelerated AC/EC). Randomisation was performed independently through the Institute of Cancer Research Clinical Trials and Statistics Unit.

Patient demographics and tumour characteristics of the different arms were compared, separately for adjuvant and neoadjuvant patients, between the randomised groups to check for any imbalance. The Kruskal–Wallis test was used for continuous or ordered categorical variables. The *χ*^2^-test or Fisher's exact test was used for comparison of binary variables.

All comparisons were two sided and, despite multiple testing, differences are reported at the 0.05 level of significance.

Comparisons were made between all patients treated with AC regimens and all patients treated with EC regimens. Comparisons were also made between all patients treated with accelerated regimens and all patients treated with standard regimens.

## Results

A total of 128 patients were randomised, and 126 analysed. Two (2%) were excluded; one withdrew consent and the other had bilirubin and transaminase levels ⩾1.5 upper limit of normal. The number randomised into each arm with their clinical and pathological characteristics is shown in Table 1. Median follow up is 23 months. Eight (6%) patients have relapsed and three (2%) have died. In 18 (14%) patients, 28 serious adverse events were recorded; 14 patients in the standard and 11 patients in the accelerated AC arms, and 1 in the standard and 2 patients in the accelerated EC arms.

### Haematological toxicity

The number of patients with grade 3/4 haematological toxicity in each subgroup (ie standard AC, accelerated AC, standard EC and accelerated EC) is shown in Table 2.

Significantly worse day 1, grade 3/4 neutropenia was seen in the standard (6 out of 61, 10%) compared to the accelerated (0 out of 65) arms, *P*=0.01. There were seven episodes of neutropenic sepsis in the standard arms (11%; all AC) and six (9%) in the accelerated arms (five AC and one EC), *P*=0.7.

No significant difference in day 1, grade 3/4 neutropenia was observed between the combined 2 and 3 weekly AC arms (3 out of 84 patients, 4%) and combined 2 and 3 weekly EC arms (3 out of 42, 7%), *P*=0.4. There were 12 (out of 84, 14%) cases of neutropenic sepsis in the AC compared with only one (out of 42, 2%) in the EC arms, *P*=0.06.

No significant difference in day 1 grade 3/4 haematological toxicity was observed between the standard AC and standard EC arms (Table 3). Similarly, no significant difference in day 1 grade 3/4 haematological toxicity was observed between the accelerated AC and accelerated EC arms (Table 3).

### Treatment delivered

Of 84 patients, 5 (6%) treated with AC received only two courses of neoadjuvant chemotherapy because of failure to respond.

Three patients were treated with three cycles, one (2%) on standard neoadjuvant AC discontinued therapy due to constipation and leg pain, and another on accelerated neoadjuvant AC developed a chest infection following three cycles. A patient on accelerated EC was treated for cellulitis and received three cycles. No significant difference in completing the intended course of chemotherapy was observed between the treatment arms (AC *vs* EC, *P*=0.6 and standard *vs* accelerated, *P*=1.0). The remaining 118 women completed four cycles.

### Treatment delays and additional growth factor support

Of 84 patients, 5 (6%) treated with AC were delayed for 1 week and 1 (1%) for 2 weeks compared to 4 patients (out of 42, 10%) delayed for 1 week in the EC arms and 1 (2%) for two weeks, *P*=0.5 (Table 2). Of 61 patients, 4 (7%) in the standard arms were delayed for 1 week and 5 (out of 65, 8%) in the accelerated arms and a further 2 in the accelerated arms were delayed for 2 weeks, *P*=0.5.

Three patients underwent dose reduction, 2 out of 42 (5%) in the standard AC arm and 1 out of 42 (2%) in the accelerated AC arm.

Of 84 patients, 13 (16%) in the AC arms required additional G-CSF compared to 4 (out of 42, 10%) in the EC arms, *P*=0.4 (Table 2). Of 61 patients, 13 (21%) in the standard arms required G-CSF support compared to 4 out of 65 (7%) in the accelerated arms who required additional G-CSF support, *P*=0.02.

### Cardiotoxicity

No patient developed clinical cardiac failure. One patient in the standard AC arm had a fall in LVEF ⩾10% to below 50%, which subsequently recovered to 53%. No significant difference in LVEF falls of ⩾10% between the AC and EC arms was observed on completing treatment (*P*=0.9), 1 (*P*=0.5) and 2 (*P*=1.0) years after therapy (Table 4). No significant difference in LVEF falls of ⩾10% between the standard and accelerated arms was observed on completing treatment (*P*=0.7), one (*P*=0.8) and two (*P*=0.8) years post therapy.

### Other toxicity

Non-haematological toxicity is shown in Table 5.

No significant difference in grade 3/4 stomatitis was observed between patients treated with both standard regimens, 3 out of 61 (5%), and both accelerated regimens, 4 out of 65 (6%), *P*=1.0. There was a non-significant trend towards more grade 3/4 stomatitis in patients treated with all AC regimens, 7 (out of 84, 8%), compared to all EC regimens, 0 out of 42 (*P*=0.09).

No significant difference in grade 3/4 stomatitis was observed between the standard AC (3/42, 7.1%) and the standard EC (0/19) arms, *P*=0.5. Similarly, no significant difference in grade 3/4 stomatitis was observed between the accelerated AC (4/42, 9.5%) and accelerated EC (0/23) arms, *P*=0.3. No significant difference in grade 3/4 stomatitis was observed between the standard (3/42, 7.1%) and accelerated (4/42, 9.5%) AC arms (*P*=1.0) or between the standard (0/19) and accelerated (0/23) EC arms.

No significant difference was observed in grade 3/4 infection rate between those treated with standard, 8 out of 61 (13%), and accelerated, 8 out of 65 (12%), regimens (*P*=1.0). Of 84 patients, 14 (17%) treated with AC developed grade 3/4 infections, including neutropenic sepsis (see above), compared with 2 (out of 42, 5%) treated with EC, *P*=0.09.

A significant difference in grade 3/4 infection was found between the standard AC (8/42, 19.0%) and standard EC (0/19) arms, *P*=0.05. No significant difference in grade 3/4 infection rate was observed between the accelerated AC (6/42, 14.3%) and accelerated EC (2/23, 8.7%) arms, *P*=0.7. Likewise, no significant difference in grade 3/4 infection rate was seen between the standard AC (8/42, 19.0%) and accelerated AC (6/42, 14.3%) arms (*P*=0.8) or between the standard EC (0/19) and accelerated EC (2/23, 8.7%) arms (*P*=0.5).

No significant difference in other non-haematological toxicity was observed between the AC *vs* EC and standard *vs* accelerated arms. There were no reports of grade 3/4 bone pain, and no patient stopped pegfilgrastim due to bone pain.

There were no treatment related deaths and no reports of myelodysplastic syndrome or acute myeloid leukaemia.

### Clinical and pathological response rates in the neoadjuvant arms

No significant difference in clinical complete and partial response was observed between the standard, 15 (79%), and accelerated AC, 16 (70%), arms (*P*=0.6). No significant difference in pCR rate was observed between the standard, 2 (11%), and accelerated AC, 3 (13%), arms (*P*=0.9).

## Discussion

This study has demonstrated that accelerated EC and AC given at 2 weekly intervals with pegfilgrastim support are at least as well tolerated as the same schedules given over standard 3-week intervals in early breast cancer, with fewer grade 3/4 neutropenia. Citron *et al* (2003) compared standard 3 weekly and accelerated 2 weekly schedules of concurrent doxorubicin and cyclophosphamide followed by paclitaxel, or sequential doxorubicin, paclitaxel and cyclophosphamide. As in our study, they found that grade 4 neutropenia was more frequent in the standard 3 weekly schedules than in the accelerated regimens (33% *vs* 6%, *P*<0.0001).

Likewise in a Phase III randomised trial comparing 3 weekly FEC with the same regimen given every two weeks with G-CSF support, a higher incidence of leucopenia was observed in the 3 weekly schedule (45 *vs* 12%, *P*<0.001; Venturini *et al*, 2005). In contrast, a higher incidence of any grade thrombocytopenia (8 *vs* 2%) and bone pain (33 *vs* 4%) was observed in the dose-dense FEC regimen.

These other trials did not look at cardiotoxicity in detail and it was therefore reassuring for us to find no evidence of increased subclinical cardiotoxicity monitored by serial LVEF with the accelerated *vs* standard approach in our study.

Our Phase II trial was not powered to detect differences in efficacy. Citron *et al* (2003) however showed a significant improvement in overall survival with dose-dense therapy (risk ratio=0.69, *P*=0.013), with 3-year overall survival of 92% in the dose-dense arms and 90% in the 3 weekly arms.

In contrast, no survival difference was found between the standard and accelerated regimens in the Phase III trial comparing 3 weekly FEC with the same regimen given every 2 weeks with G-CSF support, with an actuarial 10-year survival of 80% in the accelerated arm compared with 78% in the standard arm (*P*=0.35; Venturini *et al*, 2005). However, this trial with 1214 patients had limited statistical power to answer this question.

This exploratory Phase II trial has shown that EC, with an epirubicin dose of 90 mg m^−2^ is at least as well tolerated as AC using a doxorubicin dose of 60 mg m^−2^. The trend towards higher incidence of neutropenic sepsis, grade 3/4 stomatitis and grade 3 nausea/vomiting in the AC arms of our trial suggest that EC may be a better option and indicate the need for further comparison of the relative toxicity of doxorubicin and epirubicin. The key issue here concerns dose. A French adjuvant chemotherapy trial has shown that epirubicin 100 mg m^−2^ is more effective than 50 mg m^−2^ but also more toxic (French Adjuvant Study Group, 2001). The shape of the dose response curve for epirubicin between 50 and 100 mg m^−2^ and the optimal dose remains a topic of controversy, but 90 mg m^−2^ is also widely used and is unlikely to be significantly less effective than 100 mg m^−2^. Our observations reinforce the need for further dose response data on the efficacy of epirubicin. Interest has also focused on the use of docetaxel in combination with cyclophosphamide (Jones *et al*, 2006).

In conclusion, E(epirubicin dose of 90 mg m^−2^)C is at least as well tolerated as A(doxorubicin 60 mg m^−2^)C and accelerated AC or EC given 2 weekly with pegfilgrastim support is at least as safe and as well tolerated as when given conventionally at 3-week intervals.

## Figures and Tables

**Table 1:** Clinical characteristics of patients in the four randomised arms

	**Standard AC**	**Accelerated AC**	**Standard EC**	**Accelerated EC**
**All Patients**	***N* (%)**	***N* (%)**	***N* (%)**	***N* (%)**
Patients:	42	42	19	23
Adjuvant	23 (55)	19 (45)	19 (100)	23 (100)
Neoadjuvant	19 (45)	23 (55)		
Age: Median (Range)	48 (33–68)	50 (28–67)	50 (35–68)	56 (31–66)
				
*Menopausal status:*				
Pre	21 (50)	18 (43)	12 (63)	7 (37)
Peri	1 (2)	7 (17)	0	1 (5)
Post	17 (41)	12 (29)	5 (26)	12 (63)
Hysterectomy	3 (7)	5 (12)	2 (11)	3 (16)
				
*Receptor status:*				
ER/PR (+), HER2 (+)	4 (10)	5 (12)	2 (11)	3 (13)
ER/PR (+), HER2 (−)	13 (31)	20 (48)	11 (58)	10 (43)
ER (+), PR (−), HER2 (+)	3 (7)	1 (2)	0	0
ER (+), PR (−), HER2 (−)	5 (12)	3 (7)	0	2 (9)
ER (−), PR (+), HER2 (+)	1 (2)	0	0	1 (4)
ER (−), PR (+), HER2 (−)	1 (2)	0	0	0
ER/PR (−), HER2 (+)	2 (5)	3 (7)	3 (16)	0
ER/PR (−), HER2 (−)	11 (26)	10 (24)	2 (11)	7 (30)
	*N*=2 (5%), ER (+on biopsy/−excision), PR/HER2 (−)		*N*=1 (5%), ER/PR (−), HER2 not known	
				
*Pathology (pretreatment):*				
Infiltrating ductal	36 (86)	36 (86)	16 (84)	19 (83)
Infiltrating lobular	5 (12)	5 (12)	2 (11)	4 (17)
Other	1 (2)	1 (2)	1 (5)	0
				
*Grade:*				
I	0	2 (5)	1 (5)	1 (4)
II	18 (43)	21 (50)	9 (47)	7 (30)
III	24 (57)	18 (43)	9 (47)	15 (65)
Not known	0	1 (2)	0	0
				
*Vascular invasion:*				
Positive	6 (14)	5 (12)	4 (21)	2 (9)
Negative	31 (74)	29 (69)	15 (79)	21 (91)
Not known	5 (12)	8 (19)	0	0
				
*Pathology – adjuvant patients only*				
Pathological size (cm):	1.6 (0.7-5.0)	2.1 (.38-5.4)	1.7 (1.0-6.5)	1.9 (1.2-9.0)
				
* Nodal status:*				
Positive	3 (13)	6 (32)	2 (11)	6 (26)
Negative	20 (87)	16 (84)	17 (90)	17 (74)
Not done	0	1 (5)	0	0

**Table 2:** Grade 3/4 haematological toxicity, treatment delays and additional G-CSF in the four randomised arms

**Toxicity**	**Standard AC**	**Accelerated AC**	**Standard EC**	**Accelerated EC**	**Significance (AC *vs* EC)**	**Significance (standard *vs* accelerated)**
*Anaemia*						
Grade 3	0	3 (7%)	0	0	*P*=0.3	*P*=0.1
Grade 4	0	1 (2%)	0	0		
						
*Leucopenia*						
Grade 3	1 (2%)	0	1 (5%)	0	*P*=1.0	*P*=0.2
Grade 4	0	0	0	0		
						
*Neutropenia*						
Grade 3	2 (5%)	0	2 (11%)	0	*P*=0.4	*P*=0.01
Grade 4	1 (2%)	0	1 (5%)	0		
						
*Thromobocytopenia*						
Grade 3	0	1 (2%)	0	0	*P*=1.0	*P*=1.0
Grade 4	0	0	0	0		
						
*Treatment delays:*						
1 week	3 (7%)	2 (5%)	1 (5%)	3 (13%)	*P*=0.5	*P*=0.5
2 weeks	0	1 (2%)	0	1 (4%)		
						
*Additional GCSF:*						
	10 (24%)	3 (13%)	3 (16%)	1 (4%)	*P*=0.4	*P*=0.02

**Table 3:** Grade 3/4 haematological toxicity in the standard and accelerated AC and EC arms

	**Grade 3/4 leucopenia**	**Grade 3/4 neutropenia**	**Grade 3/4 anaemia**	**Grade 3/4 thrombocytopenia**
Standard AC	1 (2.4%)	3 (7.1%)	0	0
Standard EC	1 (5.3%)	3 (15.8%)	0	0
	*P*=0.5	*P*=0.4	*P*=1.0	*P*=1.0
Accelerated AC	0	0	4 (9.5%)	1 (2.4%)
Accelerated EC	0	0	0	0
	*P*=1.0	*P*=1.0	*P*=0.3	*P*=1.0
Standard AC	1 (2.4%)	3 (7.1%)	0	0
Accelerated AC	0	0	4 (9.5%)	1 (2.4%)
	*P*=0.1	*P*=0.1	*P*=1.0	*P*=1.0
Standard EC	1 (5.3%)	3 (15.8%)	0	0
Accelerated EC	0	0	0	0
	*P*=0.5	*P*=0.08	*P*=0.1	*P*=0.1

**Table 4:** Decrease in LVEF ⩾10% on completion, 1 and 2 years after chemotherapy.

**LVEF assessment**	**Standard AC**	**Accelerated AC**	**Standard EC**	**Accelerated EC**
End of therapy	6 (14%)	3 (7%)	2 (11%)	2 (9%)
1 year after therapy	7 (17%)	3 (7%)	0	3 (13%)
2 years after therapy	6 (14%)	3 (7%)	3 (7%)	1 (4%)
Percentage with 2-year MUGA scans	71%	69%	58%	71%

Comparison ↓ LVEF ⩾10% between AC and EC regimens.

End of therapy, *P*=0.9.

One year post therapy, *P*=0.5.

Two years post therapy, *P*=1.0.

Comparison ↓ LVEF ⩾10% between standard and accelerated regimens.

End of therapy, *P*=0.7.

One year post therapy, *P*=0.8.

Two years post therapy, *P*=0.8.

**Table 5:** Grade 3/4 non-haematological toxicities in the four arms

**Toxicity**	**Standard AC**	**Accelerated AC**	**Standard EC**	**Accelerated EC**
*Infection*				
Grade 3	6 (14%)	6 (14%)	0	2 (9%)
Grade 4	2 (5%)	0	0	0
				
*Lethargy*				
Grade 3	3 (7%)	7 (17%)	0	2 (9%)
Grade 4	0	0	0	0
				
*Nausea/vomiting*				
Grade 3	3 (5%)	2 (5%)	0	0
Grade 4	0	0	0	0
				
*Stomatitis*				
Grade 3	3 (7%)	4 (10%)	0	0
Grade 4	0	0	0	0
				
*Diarrhoea*				
Grade 3	2 (5%)	0	1 (5%)	0
Grade 4	0	0	0	0
				
*Erythema*				
Grade 3	1 (2%)	1 (2%)	0	0
Grade 4	0	0	0	0
				
*Constipation*				
Grade 3	1 (2%)	1 (2%)	0	1 (4%)
Grade 4	0	0	0	0
				
*Bone pain*				
Grade 3	0	0	0	0
Grade 4	0	0	0	0
				
*Nail changes*				
Grade 3	1	0	0	0
Grade 4	0	0	0	0
